# Enhancement of megavoltage electronic portal images for markerless tumor tracking

**DOI:** 10.1002/acm2.12411

**Published:** 2018-07-09

**Authors:** Kwang‐Ho Cheong, Jai‐Woong Yoon, Soah Park, Taejin Hwang, Sei‐Kwon Kang, Taeryool Koo, Tae Jin Han, Haeyoung Kim, Me Yeon Lee, Kyoung Ju Kim, Hoonsik Bae

**Affiliations:** ^1^ Department of Radiation Oncology Hallym University College of Medicine Seoul Korea

**Keywords:** contrast enhancement, deblurring, denoising, image quality, megavoltage electronic portal imaging device, tumor tracking

## Abstract

**Purpose:**

The poor quality of megavoltage (MV) images from electronic portal imaging device (EPID) hinders visual verification of tumor targeting accuracy particularly during markerless tumor tracking. The aim of this study was to investigate the effect of a few representative image processing treatments on visual verification and detection capability of tumors under auto tracking.

**Methods:**

Images of QC‐3 quality phantom, a single patient's setup image, and cine images of two‐lung cancer patients were acquired. Three image processing methods were individually employed to the same original images. For each deblurring, contrast enhancement, and denoising, a total variation deconvolution, contrast‐limited adaptive histogram equalization (CLAHE), and median filter were adopted, respectively. To study the effect of image enhancement on tumor auto‐detection, a tumor tracking algorithm was adopted in which the tumor position was determined as the minimum point of the mean of the sum of squared pixel differences (MSSD) between two images. The detectability and accuracy were compared.

**Results:**

Deblurring of a quality phantom image yielded sharper edges, while the contrast‐enhanced image was more readable with improved structural differentiation. Meanwhile, the denoising operation resulted in noise reduction, however, at the cost of sharpness. Based on comparison of pixel value profiles, contrast enhancement outperformed others in image perception. During the tracking experiment, only contrast enhancement resulted in tumor detection in all images using our tracking algorithm. Deblurring failed to determine the target position in two frames out of a total of 75 images. For original and denoised set, target location was not determined for the same five images. Meanwhile, deblurred image showed increased detection accuracy compared with the original set. The denoised image resulted in decreased accuracy. In the case of contrast‐improved set, the tracking accuracy was nearly maintained as that of the original image.

**Conclusions:**

Considering the effect of each processing on tumor tracking and the visual perception in a limited time, contrast enhancement would be the first consideration to visually verify the tracking accuracy of tumors on MV EPID without sacrificing tumor detectability and detection accuracy.

## 1 INTRODUCTION

Megavoltage (MV) electronic portal imaging device (EPID) has been widely used as an on‐line verification tool for treatment field in radiation therapy.^1^, ^2^, ^3^, ^4^ While the weight of the verification tool appears to be decreased after the emergence of kilovoltage (kV) imager mounted on a linear accelerator, EPID images still have their own advantages. For example, contrary to the fact that kV images are obtained from an x‐ray source which is offset 90^o^ from the treatment beam (thus always questioning the accuracy of isocenter alignment), MV EPID images are produced from the treatment beams, thus eliminating the possibility of misalignment of targets.^5^, ^6^, ^7^ Furthermore, EPID does not require additional dose when images are acquired during patient treatment.

One of the most useful applications of MV EPID is the markerless tumor tracking, in which the EPID is operated in cine mode and produces continuous portal images from the treatment beam.^8^, ^9^, ^10^, ^11^ The risk of pneumothorax from the marker implantation also prompted researchers to explore the possibility of markerless tumor tracking.^12^ While most studies on tumor tracking have been focused on tracking algorithms,^8^, ^9^, ^10^, ^11^ the importance of consecutive image display on EPID cannot be neglected. The visual confirmation of the tracking accuracy during treatment greatly facilitates action against possible misalignment of beam aperture to the moving target.

However, electronic portal images are noisy, blurred, and show poor contrast in identifying patient's anatomy in detail.^13^, ^14^, ^15^ Image enhancements have been used to obtain the optimal readability of EPID images, including contrast improvement, edge deblurring, and noise reduction.^16^, ^17^, ^18^ A sequential application of these three image processing protocols was also proposed.^18^ However, image processing during tumor tracking requires consideration of the time needed since the image should be processed without interfering with the next incoming image. Therefore, the most effective enhancement of EPID images is needed within a limited time. An effective algorithm showing several desirable improvements within a limited time is the best option. However, at first, comparative analysis of image processing results from each representative algorithm is an advantage.

Both unsharp masking for deblurring and contrast improvement have been the most widely used algorithms in medical imaging applications including noise reduction. In this study, therefore, these algorithms were individually applied on EPID portal images and the effect of each algorithm on image visualization was compared. In reality, contrast‐limited adaptive histogram equalization (CLAHE) for contrast improvement and total variation (TV) deconvolution for unsharp masking were adopted.^16^, ^19^, ^20^, ^21^, ^22^, ^23^ A median filter was employed for noise reduction. In addition, we were interested in determining whether the modified images affect the tumor auto‐detection capability during tumor tracking. Therefore, by implementing a mask‐based tracking algorithm, the tumor detection accuracy was also compared.

## 2 METHODS

MV EPID aS‐500 imager attached to a Varian 21 EX (Varian Medical Systems, Palo Alto, CA, USA) was used with a 6 MV beam. Images with a resolution of 512 × 384 and pixel size of 0.784 mm were obtained. For image processing evaluation, the image of the QC‐3 quality phantom (Standard Imaging, Middleton, WI, USA) placed at a distance of 35 cm from the EPID surface with the source to EPID distance of 140 cm was acquired. Furthermore, a single patient's setup image receiving whole brain treatment was processed to assess the clinical usefulness. To find out the effect of the enhanced images on the tumor tracking accuracy, two lung cancer patients’ cine images were obtained during radiation treatments.

Human visual perception is known to be affected the most by contrast changes.^16^, ^17^, ^24^ In this study, CLAHE algorithm was used for contrast enhancement.^16^, ^21^, ^23^ The global histogram equalization treating the whole image occasionally yields an indiscernible result with a flat histogram. To remedy this drawback, adaptive histogram equalization (AHE) divides the image into subsections and applies histogram equalization to each subsection. However, it has a tendency to emphasize local histogram excessively and increases noise. CLAHE is the addition of the contrast limitation in AHE. The algorithm implemented in Matlab (r2010, MathWorks, MA, USA) was used with default 8 × 8 subsections.

Unsharp masking has been widely used for image deblurring in many applications. However, in principle, it does not restore the unblurred ideal image. It just modifies the image boundary by adding the mask which is generated by subtraction of the blurred image from its original, resulting in a shift of the sharp boundary from the original position.^25^ In general, the observed image **g** is modeled by **g = Hf + η** in matrix form, in which **f** is the ideal image, and **η** is the noise added. The point spread function (PSF) **H** containing various deblurring effects is convolved with the ideal image. To recover the image without blur, the problem is not well‐defined. Looe et al., utilized iterative deconvolution to get a deblurred image by limiting the iteration based on a consecutive refined image difference.^15^ However, regularization method has been widely studied and established for the ill‐defined problem, and the total variation is one of them.^19^, ^20^, ^22^ Therefore, to obtain the real deblurred image preserving sharp edges, total variation deconvolution was adopted and the following was minimized,

(1) $${\left\|\mathrm{Hf}-g\right\|}^{2}+{\left\|f\right\|}_{\mathrm{TV}}\;\;\text{with}\;\;{\left\|f\right\|}_{\mathrm{TV}}=\Sigma \sqrt{\beta_{x}^{2}\left[\right.{\left[D_{x}f\right]}^{2}+\beta_{y}^{2}\left[\right.{\left[D_{y}f\right]}^{2}}$$

in which μ is a regularization parameter, βs are control parameters, and Ds are gradient operators. Additional details can be found in the reference.^22^ The required 2D PSF was borrowed from the results of aS‐1000 EPID notwithstanding different resolution of 1024 × 1024 with 0.4 mm pixel size, in which the Lorentzian function in normalized form $\frac{1}{{\left(1+\frac{x^{2}+y^{2}}{\lambda^{2}}\right)}^{3/2}}$ was suggested with a representative parameter value of λ = 0.5 for *E* = 6 MV.^15^ Additionally, to examine the effect of reduced noise, a median filter for 3 × 3 pixels was also applied to original image set.

**Figure 1:**
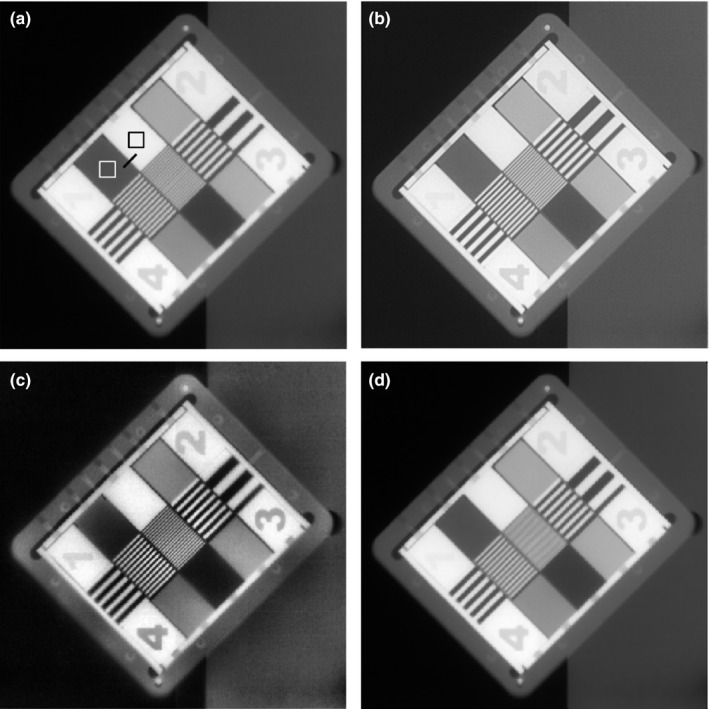
EPID images of QC‐3 phantom: (a) original, (b) deblurred, (c) CLAHE, and (d) denoised. SNR and contrast were measured in both boxes drawn and blurriness was measured along the line profile in (a).

To assess the image quality of QC‐3 phantom, three parameters of contrast value, signal‐to‐noise ratio (SNR), and blurriness were introduced. The contrast value was calculated as follows: $\text{Contrast}=\frac{I_{w}-I_{b}}{I_{w}}$, where *I*_w_ and *I*_b_ were the average intensities within a 10 × 10 square pixels inside a white rectangular and a black rectangular, respectively, between two numbers ‘1’ and ‘2’ of the phantom image (Fig. 1(a)).^26^ The signal‐to‐noise ratio (SNR) was evaluated using the following formula: $\text{SNR}=\frac{I_{w}}{\sigma_{b}}$, where *I*_w_ was the same as above, and the σ_b_ was the standard deviation inside the same black box also mentioned above. The blurriness was evaluated from the tilted line profile drawn in the Fig. 1(a). After fitting the measured profile data using a sigmoid‐like Boltzman function, the width of 10–90% values between maximum and minimum was calculated. Therefore, the smaller the width is, the less blurred the image is. The unit was not exactly in pixel number due to the oblique line profile and considered as relative values. All results were normalized to those of original.

**Figure 2:**
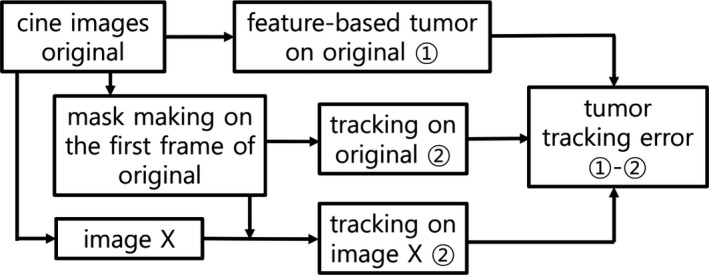
Schematic work flow for the evaluation of tumor tracking accuracy (X = deblurred, CLAHE, and denoised).

**Figure 3:**
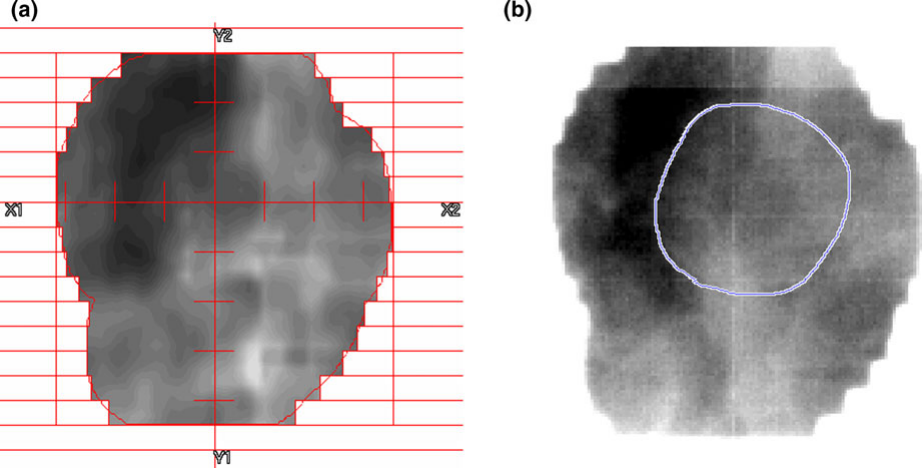
(a) Example of DRR and (b) its corresponding portal image with mask for tumor tracking.

We experimented tumor tracking in each image set (original, deblurred, CLAHE, and denoised) to explore the effect of image modification on tracking accuracy. Overall, the tracked tumor position for each frame was measured with reference to the manually selected feature point which was common to four corresponding frame images. Fig. 2 depicts a simplified work flow. Initially, a feature‐based tumor position was determined for all original cine images. To this end, three feature points specifying tumor position for each original image were manually selected and the average point was regarded as the positional origin for that frame in all image sets (*T*_m_ (*k*): manually selected origin for frame *k*). Second, with reference to tumor in digitally reconstructed radiographs (DRR), a region of interest (ROI) specifying the tumor was drawn (Fig. 3) on the first frame of original images for each fraction and for each beam, and, a binary mask was created corresponding to the ROI. Third, the mask‐based tumor tracking was fulfilled for each image set. To take a procedure example using the original image set, the first frame represents the reference image. For the automatic detection of tumors on subsequent frames, the mask was moved pixel‐by‐pixel within a fixed region of 10 × 10 square pixels (7.8 × 7.8 mm^2^), in which the tumor motions were pre‐examined and assured to be within the defined region. As a tracking algorithm, the minimum position of the mean of the sum of squared pixel differences (MSSD) between the first (reference) and the objective frame was taken as the best matching point (*T*_t_ (*k*): tracked tumor position for frame *k*).^8^, ^27^ The same procedures were repeated using deblurred, CLAHE and denoised sets. Fourth, with the feature‐based position as the origin, the mask‐based tumor position for each frame was calculated ($d\left(k\right)=T_{m}\left(k\right)-T_{t}\left(k\right)$: relative distance vector of tumor position for frame *k*). After correction of the relative distance vector of the reference frame, the mask‐based tumor tracking accuracy was obtained (*e*(*k*) = *d*(*k*) − *d*(0); ‘0’ is the reference). The comparison of this accuracy for four image sets (original, deblurred, CLAHE, and denoised) revealed the tracking accuracy variation based on image modification.

## 3 RESULTS

The original EPID image of QC‐3 resolution phantom is shown in Fig. 1(a). TV regularized deblurred image and CLAHE processed one are shown in Fig. 1(b) and 1(c), respectively. Due to insufficient pixel resolution of aS‐500 EPID, edges of structures including stripes with varying thickness are rather coarse. However, image processing effects can be clearly recognized. Deblurred image shows resolution enhancement which ensures adequacy of the PSF model that was adopted. Object visibility for fine details is increased. However, the contrast is almost similar to that of the original case. Therefore, notable improvement in image identification is not observed. Contrast is much improved in the case of CLAHE. For example, figure ‘1’ at the left corner of the phantom is easily recognized. The reduced noise case is shown in Fig. 1(d), in which the preexistent details are diminished with a worse resolution. The central fine stripes are barely discernible.

The assessments for image quality in QC‐3 phantom are collected in Table 1. First, the SNRs of deblurred and CLAHE are decreased and the CLAHE the most, which reflects the decrease of image uniformity. In case of blurriness, the deblurred image shows the sharpest boundary as expected. However, the blurriness evaluation was not simple both for CLAHE and denoised. The selected metric calculating the 10–90% width at the boundary of black and white box failed to reflect the fogged fine stripes in the central part of denoised image in Fig. 1(d). Based on the line pairs per centimeter, the central part of the denoised shows the worst sharpness. For contrast, CLAHE was not outperformed, and others maintained existing levels of contrast.

**Table 1:** QC‐3 phantom image characteristics

	SNR	Blurriness	Contrast
Original	1.00	1.00	1.00
Deblurred	0.43	0.45	1.00
CLAHE	0.01	1.72	31.67
Denoised	1.95	1.01	1.00

**Figure 4:**
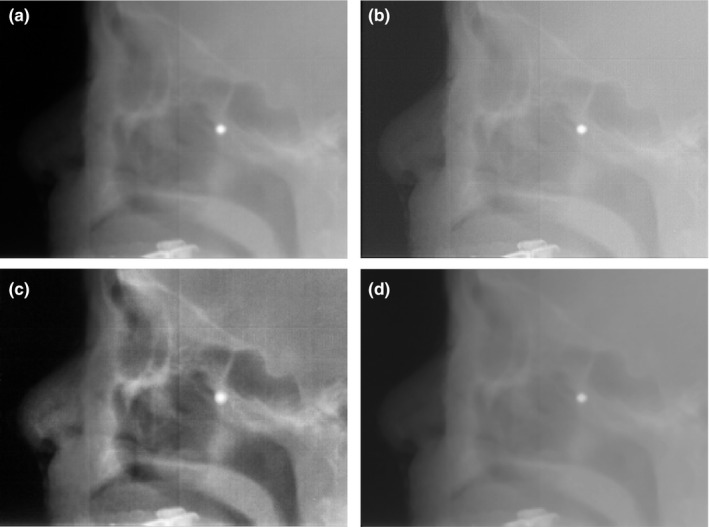
EPID setup images for whole brain treatment: (a) original, (b) deblurred, (c) CLAHE, and (d) denoised.

As an example of clinical application, the lateral EPID setup image for the whole brain treatment was processed in Fig. 4. The central small white circle is the physical port film graticule. The structures including maxillary sinus and sphenoidal sinus are vague in the original image, which is a typical feature of MV images. Deblurred result is displayed in Fig. 4(b) and shows reduced blurriness. However, the overall image quality after the deblurring procedure is similar to that of the original, and visual interpretation is slightly improved. CLAHE processed image shown in Fig. 4(c) has distinct features, thus providing easier identification of the anatomy. The denoised image in Fig. 4(d) is soft and the object boundary is not clear.

**Figure 5:**
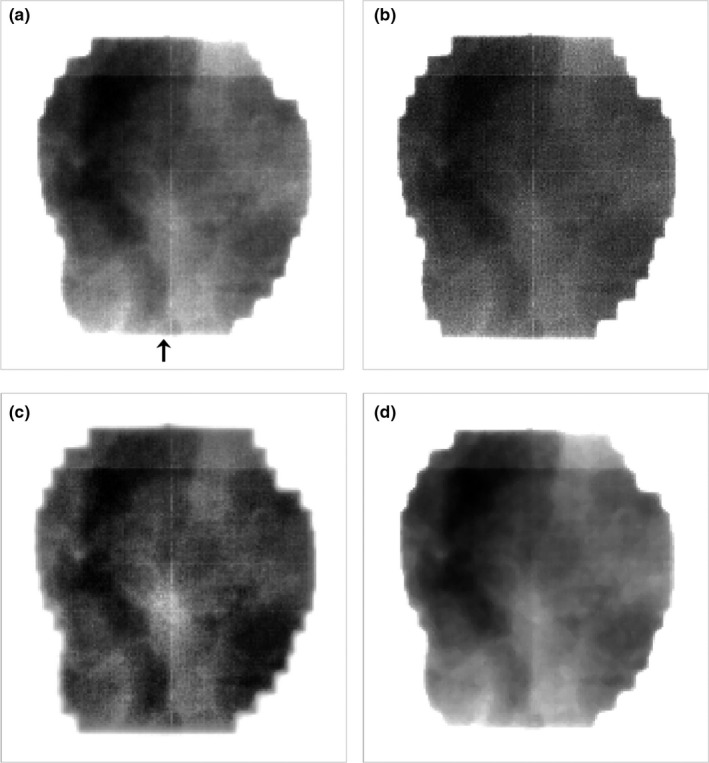
EPID cine‐mode images of a lung cancer patient undergoing treatment: (a) original, (b) deblurred, (c) CLAHE, and (d) denoised.

Fig. 5 shows an example of portal image obtained from a lung cancer patient under treatment. As already seen in the whole brain case, the contrast‐enhanced case of Fig. 5(c) reveals a definite tumor shape and nearby anatomy. The vivid contrast facilitates, for example, to verify tumor tracking accuracy at a glance unlike others.

**Figure 6:**
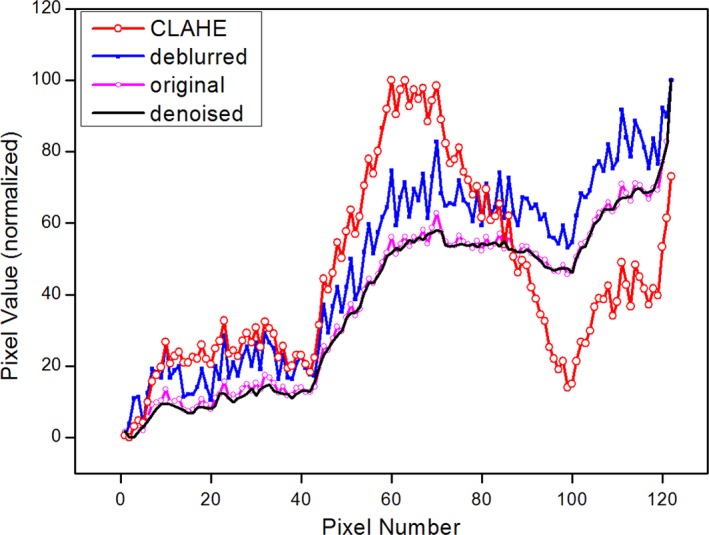
Comparison of pixel value profiles of the original, deblurred, CLAHE, and denoised lung patient's images. These profiles were obtained along the arrow in Fig. 5(a).

In Fig. 6, the longitudinal profiles through the arrow marked in Fig. 5(a) recapitulate the same impression in the processed images, in which the maxima and the minima are matched with the same values to achieve a fair comparison. The profile of deblurred image behaves similar to that of the original with slight improvement which is expected from the processed image. More specifically, the profile of deblurred images shows moderately enhanced peak‐to‐peak pattern. The noise reduced one clearly shows smooth behavior, and as already seen in the phantom case, the details are wiped out. Meanwhile, CLAHE result is very impressive. It manifests objects‐like features clearly from the tumor and nearby structures. Contrast enhancement increases the readability of EPID image resulting in an informative outcome.

A total of 80 EPID frames were acquired under cine mode during treatment. Except for five frames for the references, 75 images were tried as objects for the mask‐based tumor detection. Among them, five original frames and the same five denoised ones were not detected. Among the five undetected images, three yielded tumor position in deblurred set, resulting in two undetected. In the case of CLAHE, the target position was determined for all images. The undetected tumor location was due to multiple minimum points, which may be attributed to their weak contrast with similar pixel values inside the mask search region. The tumor detection may depend on the tracking algorithm and also on the mask shape in our case. Therefore, the slightly higher detection power in CLAHE may not be definitive and needs further confirmation. The detection error was measured as the distance between the target and the manually selected feature‐based ground truth (Table 2). The overall accuracy is around 3 mm. However, deblurring improved the detection accuracy, and denoising resulted in worse error. CLAHE presents almost the same accuracy as that of original.

**Table 2:** Mask‐based tumor detection errors (mm).^a^

	Superior–Inferior	Left–Right
Original	3.0 (4.4)	2.7 (3.2)
Deblurred	2.8 (4.2)	2.6 (3.2)
CLAHE	2.9 (4.4)	2.7 (2.9)
Denoised	3.2 (4.5)	3.1 (3.4)

a Mean distance from feature‐based origin with standard deviation.

## 4 DISCUSSION

In general, images obtained from devices are corrupted by noise and blurriness. Furthermore, images of MV EPID show poor contrast. For noticeable image improvement in EPID, it has already been suggested that a multistep image processing consisting of contrast enhancement, noise reduction and edge sharpening should be applied.^18^ Diez et al. have improved the contrast of EPID images by introducing a combination of image manipulation algorithms, e.g., an inverse restoration filter and a local contrast enhancement.^28^ Meanwhile, if the visual confirmation of the tumor tracking is straightforward, it is a great help for accurate beam delivery. However, for real‐time application of EPID images in tumor tracking, the required processing time needs to be minimized and therefore, strategic approach is needed. Here, the ‘real‐time’ refers to image processing completed and displayed without interfering with the next incoming image under cine mode.^10^ The purpose of this study was to determine the enhancement resulting in substantially significant results for clinical application such as tumor tracking.

Deblurred images of the phantom and patients (Fig. 1(b), Fig. 4(b) and Fig. 5(b)) display enhanced edges and demonstrate that our deblurring approach worked properly. However, as shown in Fig. 6, profile comparison reveals that deblurred images are not so impressive since these images do not show distinct and immediate differences from the originals. Noise reduction, also one of the common image processing algorithms, fulfilled its role as expected. However, the loss of details in the QC‐3 phantom questions its usage in clinical practice because the image should contain as much information as possible for clinical decision‐making such as patient setup procedure. Meanwhile, CLAHE results shown in Fig. 1(c), Fig. 4(c) and Fig. 5(c) are captivating. The enhanced contrast makes these images very rich in structures and useful for clinical applications. The pixel value profile of CLAHE shown in Fig. 6 clearly demonstrates enhanced readability again. However, amplification of noise is also observed. Noise has been reported to be increased slightly during contrast improvement and deblurring, and incorporation of another filter was suggested to limit artifacts.^29^, ^30^ In our study, however, the increase of noise was neglected to simulate the minimal processing time, and noise influence on image interpretation was marginal.

The superior‐inferior (SI) and left‐right (LR) tumor detection errors between manual and tracking in this study are larger than those of Anne et al., in which they adopted the same tracking algorithm as ours; however, the means with standard deviations in SI and LR were 1.0 ± 1.1 mm and 0.6 ± 0.6 mm, respectively.^8^ The different accuracy may be attributed to their different approach for the manual determination of the reference point. They utilized the same tracking mask for manual detection by adjusting the mask position manually to cover the target to the maximum extent. Meanwhile, our feature‐based point selection focused on local characteristics. However, this factor was not the focus of our experiment since we merely sought a reference point to calculate the relative position of the tumor as long as the reference point was selected consistently in the original image set.

The increased tumor detectability and comparable accuracy of CLAHE are unexpected because the modified image appears to overemphasize tumor and nearby tissues, resulting in discrepancies of the image from the usual portal images. However, considering that tumor detection is a relative operation, the result is understandable. Since the tracking is based on the comparison between the reference image and the object image, as long as two images are modified using the same operation, both can be considered equivalent to the original images in the detection process. Therefore, the single contrast improvement facilitates users in verifying the tumor position inside the proper beam aperture without trade‐off in both power and accuracy of the detection during markerless tumor tracking. In this study, we did not thoroughly investigate the tracking algorithm itself, and its limitations are described in the reference.^27^ However, we should mention one thing about the tracking accuracy in this study that the minimum position from MSSD for each frame was possibly influenced by the mask search region.

The detection accuracy was increased for deblurred set, and decreased for the denoised set. Yip et al., implemented a tracking algorithm (called STiL) based on matching of automatically detected multiple landmarks between the reference and the object image.^10^, ^11^ Their algorithm was reported to enhance the tracking accuracy compared with single template matching. They investigated the correlations between tumor tracking accuracy and blurriness, noise, and contrast by varying the number of frames to obtain the averages. The results were that the accuracy increased by decreasing blurriness and increasing contrast. The relation with noise was not clear in the patient's images. These results are largely consistent with our findings except that noise was negatively correlated with detection accuracy in our study. The tracking algorithm in our study was based on pixel intensity and known to be suitable for low‐contrast images.^27^ Consistency in results from two different algorithms increased the reliability of our results.

We presumed that all the acquired images under cine mode should be processed with a better visibility. Toward this end, contrast modification might be the first option for high‐frequency frame images. One of the recent studies utilized 12.87 frames per second for EPID‐based tumor tracking, and also introduced a prediction process to compensate the delay time from image acquisition and tumor detection to adjustment of the treatment machine for accurate targeting.^11^ It can be questionable whether all frames should be processed for visual perception. However, recognizing that visual verification of the tracking accuracy helps physicians/technicians intervene against possible irregularity, processing all frames is beneficial for prompt response. Of course, the automatic beam‐off can be considered for the unexpected event.

Following this experiment, the effectiveness of total variation regularized deblurring was questioned for rapid image processing, which facilitates other applications of EPID image such as patient setup procedure since the setup accuracy can be affected by the detailed anatomy. However, repeated instant confirmation of tracking accuracy seems not to require the image detail. Therefore, unsharp masking, e.g., may be adequate even with its boundary shift mentioned above in the introduction. This problem of balance between image quality and effectiveness warrants further study. There are countless algorithms for contrast improvement, deblurring, and denoising. In the design of this study, we did not optimize or investigate the most effective or the fastest algorithm in a limited time interval between subsequent frames of tumor tracking. Therefore, our methods may not be the best choice for image enhancement for tumor tracking.

One of the limitations in this study is the limited number of images needed to explore the diverse image characteristics. For example, the tumor status depending on whether it is isolated or adjacent to nearby organs may result in varying detection accuracy of the modified cine images. Furthermore, the tracking algorithm may lead to a specific result. Therefore, various tracking algorithms such as mutual information and matching of multiple landmarks need to be tested for various image data.^10^, ^31^

During patient treatment with EPID‐based tumor tracking, in which cine images are input and displayed subsequently with a relatively high frequency, the improved visual identification of the moving target greatly facilitates accurate treatment. Contrast enhancement appears to be the primary technique for poor‐quality MV EPID images. Deblurring, if allowed, can add details to the image. The combination of algorithms would contribute to visual verification and detection accuracy if the processing can be completed without interfering with the subsequent image.

In summary, visual identification of the moving target during tumor tracking greatly facilitates accurate beam delivery. While deblurring made MV EPID image rich in detail, contrast enhancement dramatically increased the visual perception of MV portal image. When deblurred image set increased the detection accuracy in tumor auto tracking, contrast enhanced image set, at least, maintained the accuracy of the original image. Therefore, contrast enhancement should be the primary option for time‐sensitive applications. For routine setup process, the combined process of deburring and contrast modification would be advantageous.

## 5 CONCLUSIONS

The poor quality of MV EPID image requires efforts for visual verification of the tumor location under tracking treatment. Image processing during tumor tracking requires completion and display in a limited time interval between successive incoming frames. Therefore, we investigated the individual contribution of deblurring, contrast improvement and denoising on the MV EPID image. After processing, deblurring enhanced the details of the image, whereas, denoising made the image clean at the cost of image resolution. Contrast processing greatly improved the total impression on EPID images and facilitated anatomical investigation. The combined operation of contrast enhancement and deblurring would markedly improve the readability of MV portal image. Furthermore, we implemented and examined the intensity‐based tumor tracking. Out of a total of 75 images, both original and denoised sets failed to determine the tumor position of the same five images. Among those undetected five, deblurred set failed to determine the two cases. Only contrast enhancement resulted in tumor detection of all the images. Detection accuracy was increased by deblurring of images compared to original image set. Conversely, denoised case decreased the accuracy. In the case of contrast improvement, the tracking accuracy was similar to that of original set. Considering the effect of each processing on tumor auto tracking and visual perception in a limited time, contrast enhancement may be the first step and the most effective option.

## CONFLICT OF INTEREST

No conflicts of interest.
